# Water extract of *Clinacanthus nutans* leaves exhibits in vitro, ex vivo and in vivo anti-angiogenic activities in endothelial cell via suppression of cell proliferation

**DOI:** 10.1186/s12906-018-2270-1

**Published:** 2018-07-06

**Authors:** Chin Theng Ng, Lai Yen Fong, Jun Jie Tan, Nor Fadilah Rajab, Faridah Abas, Khozirah Shaari, Kok Meng Chan, Fariza Juliana, Yoke Keong Yong

**Affiliations:** 1 Physiology Unit, Faculty of Medicine, AIMST University, 08100 Bedong, Kedah Malaysia; 2 Department of Pre-clinical Sciences, Faculty of Medicine and Health Sciences, Universiti Tunku Abdul Rahman, 43000 Kajang, Selangor Malaysia; 3 Advance Medical and Dental Institute, Universiti Sains Malaysia, Penang, Malaysia; 4 Faculty of Health Sciences, Universiti Kebangsaan Malaysia, 50300 Kuala Lumpur, Malaysia; 5 Department of Food Science, Faculty of Food Science and Technology, Universiti Putra Malaysia, 43400 UPM Serdang, Malaysia; 6 Faculty of Science, Universiti Putra Malaysia, 43400 UPM Serdang, Malaysia; 7 Department of Human Anatomy, Faculty of Medicine and Health Sciences, Universiti Putra Malaysia, 43400 UPM Serdang, Selangor Malaysia

**Keywords:** *Clinacanthus nutans*, Acanthaceae, Angiogenesis, Chick embryo choriollantoic membrane, Aortic ring

## Abstract

**Background:**

*Clinacanthus nutans* (Burm. f.) Lindau. has traditionally been using in South East Asia countries to manage cancer. However, scientific evidence is generally lacking to support this traditional claim. This study aims to investigate the in vitro, ex-vivo and in vivo effects of *C. nutans* extracts on angiogenesis.

**Methods:**

*C. nutans* leaves was extracted with 50–100% ethanol or deionised water at 1% (*w*/*v*). Human umbilical veins endothelial cell (HUVEC) proliferation was examined using MTT assay. The in vitro anti-angiogenic effects of *C. nutans* were assessed using wound scratch, tube formation and transwell migration assays. The VEGF levels secreted by human oral squamous cell carcinoma (HSC-4) cell and HUVEC permeability were also measured. Besides, the rat aortic ring and chick embryo chorioallantoic membrane (CAM) assays, representing ex vivo and in vivo models, respectively, were performed.

**Results:**

The MTT assay revealed that water extract of *C. nutans* leaves exhibited the highest activity, compared to the ethanol extracts. Therefore, the water extract was chosen for subsequent experiments. *C. nutans* leaf extract significantly suppressed endothelial cell proliferation and migration in both absence and presence of VEGF. However, the water extract failed to suppress HUVEC transmigration, differentiation and permeability. *C. nutans* water extract also did not suppress HSC-4 cell-induced VEGF production. Importantly, *C. nutans* water extract significantly abolished the sprouting of vessels in aortic rings as well as in chick embryo CAM.

**Conclusion:**

In conclusion, these findings reveal potential anti-angiogenic effects of *C. nutans*, providing new evidence for its potential application as an anti-angiogenic agent.

## Background

Angiogenesis is the main contributor to the transition of pre-invasive and dormant tumour cells to a more invasive and malignant cells, through establishing additional blood vessel network from pre-existing vasculature to enable more efficient oxygen and nutrient delivery for growth. Such pathologic event is orchestrated by overexpression of vascular endothelial growth factor (VEGF) secreted by tumour cells, the known factor that promotes endothelial cell proliferation, invasion, migration and capillary tube formation [1]. Hence, inhibition of tumour angiogenesis by targeting VEGF pathway has emerged as an important strategy in combating cancers [2].

However, the new blood vessels formed are unlike normal blood vessels; they exhibit increased permeability, and cause diminished blood flow and create hypoxic microenvironment within the tumour [3]. To date, not more than 15 identified anti-cancer drugs that target tumour angiogenesis are granted approval from US Food and Drug Administration. In addition to undesirable side effects and low patient survival, those agents often couple with low therapeutic efficacy and high risk of drug resistance after long-term treatment, as reported in trials involving small cell lung cancer patients treated with bevacizumab and sunitinib [4]. The current anti-angiogenic drugs are single-target based agents which often could not achieve desired outcomes from inhibiting angiogenesis, mainly because of the redundancy in the angiogenic pathways.

Traditional plant extracts are source of multi-targeted therapeutic agents as they contain various medicinal phytochemicals. The production cost is usually low due to their abundance in nature. Plant extracts may also reduce the risk of adaptive resistance that is commonly seen in therapy with single agent [3]. *Clinacanthus nutans* (Burm. f.) Lindau. is locally known as Sabah Snake Grass, belonging to the Acanthaceae family. *C. nutans* has been used traditionally as a medicinal herb in tropical Asia to treat various diseases such as insect bites, diabetes mellitus, diuretics and fever [5]. Previous studies have reported that *C. nutans* possesses cytotoxicity [6], anti-proliferative, anti-oxidant [7] and anti-inflammatory [8] activities. Ethanol extract of *C. nutans* has been shown to impede hepatoma in mice through induction of apoptosis and enhancement of immune response [9]. Researchers have also demonstrated that *C. nutans* methanol extract induces human melanoma cell apoptosis [10]. Despite claims regarding the use of *C. nutans* in treating various cancers, the anti-angiogenic potential of *C. nutans* has never been carefully examined. Here we showed that *C. nutans* is a potential anti-cancer agent that possesses inhibitory effects on VEGF-mediated angiogenic events, based on in vitro findings using human umbilical vein endothelial cells (HUVECs), ex vivo test using rat aortic ring and in vivo investigation using chick embryo chorioallantoic membrane (CAM) assays*.*

## Methods

### Chemicals and reagents

HUVECs, EndoGRO culture media, human VEGF_165,_ Millicell cell culture inserts with pore size of 8.0 μm, in vitro vascular permeability assay kit and EndoGRO-LS complete culture media kit were purchased from Milipore. Growth factor-reduced matrigel was purchased from BD Bioscience. Human oral squamous cell carcinoma (HSC-4) cells and Dulbecco’s Modified Eagle’s Medium (DMEM) were purchased from American Type Culture Collection (ATCC). Human VEGF Quantikine ELISA kit was purchased from R&D Systems. Suramin (purity> 99% by TLC) was purchased from Sigma-Aldrich.

### Plant material

Whole plant of *Clinacanthus nutans* (Burm. f.) Lindau was harvested from the Sendayan Commodities Development Centre in Seremban, Negeri Sembilan, Malaysia. The plant was verified by Dr. Shamsul Khamis, botanist at Institute of Bioscience, Universiti Putra Malaysia. The specimen of *C. nutans* has been deposited at the herbarium of the Institute of Bioscience, Universiti Putra Malaysia (voucher number: SK 2883/15).

### *C. nutans* leaf extraction

*C. nutans* leaf extracts were obtained from our previous work [11]. Briefly, the plant was harvested, cleaned with water, dried and separated into leaves and stems. The leaves were then dried at room temperature under the shade in a well-ventilated room and ground to fine powder. Next, the ground powder was extracted by sonicating in 50, 70, and 100% ethanol or deionised water at 1% (*w*/*v*) for 1 h without heating. The extraction was repeated thrice for each sample, followed by filtered-sterilization and vacuum evaporation to dry and freeze-dry the extracted samples prior to storing at 4 °C until further analysis.

### Cell culture

HUVECs were purchased from Merck, Malaysia, and grown in T-25 cell culture flasks with EndoGRO culture media. HUVECs between passage three to six were used in the experiments. Human oral squamous cell carcinoma (HSC-4) cells from American Type Culture Collection (ATCC) were maintained in Dulbecco-modified Eagle Medium (DMEM) supplemented with 10% FBS and 4 mM L-glutamine. All the cells were incubated at 37 °C in an incubator with 95% humidified air and 5% CO_2_.

### Cell proliferation assay

Cell proliferation assay was performed as described previously by Mosmann [12], with some modifications. HUVECs were plated onto 96-well plates at a cell density of 1.0 × 10^4^ cells per well. After 24 h, cells were treated with different *C. nutans* extracts and subsequently incubated for 24, 48 and 72 h in separate well plates. After indicated times, 10 μl of MTT (5 mg/ml stock concentration) was added into each well and incubated in an incubator for additional 4 h. Then, MTT solution was removed and the purple formazan was dissolved in 100 μl of dimethyl sulfoxide (DMSO). The absorbance was measured using a microplate reader (Tecan M200 Infinite) at a wavelength of 570 nm with a reference wavelength of 650 nm.

### Wound healing assay

The assay was carried out according to Kavitha et al. [13] with some modifications. HUVECs were plated onto 6-well plates and allowed to grow until confluence. Then, a defined scratch gap was created using a 100–200 μl-pipette tip. All detached/dead cells were removed by several washes with media. Then, cells were co-treated with human VEGF_165_ and *C. nutans* extracts. Images were captured at 40× magnification, at 0 h (baseline) and 12 h as soon as the gap in VEGF-treated groups was completely covered by cells, using an inverted microscope (Olympus CKX31). The cell migration was quantified using the formula: Initial wound distance minus final wound distance divided by two [13].

### Tube formation assay

Tube formation method was performed according to Arnaoutova and Kleinman [14]. Briefly, growth factor-reduced Matrigel™ was pipetted into pre-chilled 96-well plates and polymerized at 37 °C for 30mins. HUVECs (1.5 × 10^4^ cells per well) were seeded onto the Matrigel-coated plates. *C. nutans* extract at 50 to 1000 μg/ml were added at plating. After 3 h, the tubular structure was visualized and the images were captured using an inverted microscope at 40× magnification. The tube length was quantified using an Image J with integrated angiogenesis analyser plugin (Gilles Carpentier, Faculté des Sciences et. Technolo-gie, Université Paris Est, Creteil Val de Marne, France).

### Transwell migration assay

Transmigration of HUVECs was studied as described previously by Kavitha et al. [13] with slight modifications. Briefly, HUVECs (3 × 10^4^ cells per well) were cultured on Millicell cell culture inserts with pore size of 8.0 μm, and treated with 50 to 1000 μg/ml *C. nutans* extract dissolved in EndoGRO media supplemented with 0.5% FBS. Bottom wells were filled with 750 μl of EndoGRO media containing 10 ng/ml human VEGF_165_ and extract similar to that of the inserts. After 6 h, non-migrated cells on the insert membrane were removed by cotton swabs, and migrated cells were fixed with 4% paraformaldehyde and stained with 0.1% crystal violet. The dyes were extracted using 10% acetic acid and the absorbance was measured by a microplate reader at 590 nm.

### In vitro vascular permeability assay

The in vitro vascular permeability assay was performed according to manufacturer’s protocol. Briefly, HUVECs were grown to confluence on collagen-coated inserts. Next, the monolayers were treated with different concentrations of *C. nutans* extract. After 24 h, FITC-dextran solution was added onto the cultured HUVEC monolayer for 20 min, and the fluorescence intensity of FITC-dextran that crossed the cell layer were measured using Tecan Infinite M200 fluorescence plate reader at 485/530 nm (Excitation/Emission), as described previously by Ng et al. [15].

### VEGF levels

HSC-4 cells were seeded onto 96-well plates at a cell density of 1 × 10^4^ cells per well. Then, cells were treated with 50 to 1000 μg/ml of *C. nutans* extract for 72 h. VEGF levels in HSC-4 were quantified using Human VEGF Quantikine ELISA kit. Absorbance were read at 450 nm and corrected to 570 nm.

### Experimental animal

Healthy male Spradue Dawley rats (aged 6–8 weeks old) from the Faculty of Medicine and Health Sciences, Universiti Putra Malaysia, were acclimatized for 7 days under standard environmental conditions. The rats were free access to standard laboratory chow and water ad libitum, and were kept in a room with 12 h day/night cycle.

### Ex-vivo aortic ring assay

The animals were euthanatized by CO_2_ exposure and aortas were isolated from male *Sprague Dawley* rats (UPM/IACUC/AUP-R071/2015) according to Bellacen and Lewis [16]. Pre-chilled 48-well plates were filled with 150 μl of Matrigel™ and allowed to polymerise for 30 mins at 37 °C. Rat aortas were rinsed with cold sterile phosphate-buffered saline and cut into 1 mm-long cross sections. Aortic rings were placed on Matrigel and covered with an additional 150 μl of Matrigel. The aortic rings were fed with 500 μl of complete culture media, or supplemented with *C. nutan* at 50 to 1000 μg/ml. The treatments were replaced daily. Growing sprouts, at day 8, were photographed with an inverted microscope (Olympus), and the sprout length was analysed by Image J NIH software program.

### In vivo chick embryo chorioallantoic membrane (CAM) assay

Fertilized chicken eggs were purchased from a poultry farm (Hing Hong Sdn. Bhd) and incubated in a 38 °C oven with 60% humidified air for 72 h. The CAM assay was performed according to West et al. [17]. Briefly, the shell of eggs was gently cleaned and wiped with 70% ethanol. With caution, a small hole was created with an 18-gauge needle on the narrow end of the eggs and 3 ml of albumin was removed. The needle hole was then sealed with clear tapes. Subsequently, a small window was carefully created on the top-most surface of the eggshells. The windows were then covered by sterile parafilm and the eggs were placed back into the oven. After 8 days, 500, 1000 and 2000 μg/ml of *C. nutans* were applied onto sterile filter disks and dried before grafting on the CAM. After that, the windows were sealed by a sterile parafilm and the eggs were placed back into the oven. After 48 h, the CAMs were fixed with 4% paraformaldehyde at 4 °C, overnight. Angiogenesis levels were determined by counting the number of vessels contacted to *C. nutans* disks.

### Statistical analysis

The data were expressed as the mean ± SEM. Statistical analysis was performed using IBM SPSS 20.0. One-way analysis of variance (ANOVA) followed by Tukey’s test was used to compare means for the multiple groups. *p* values less than 0.05 (*p* < 0.05) were considered to be statistically significant.

## Results

### *C. nutans* water extract suppresses endothelial cell growth

**Fig. 1:**
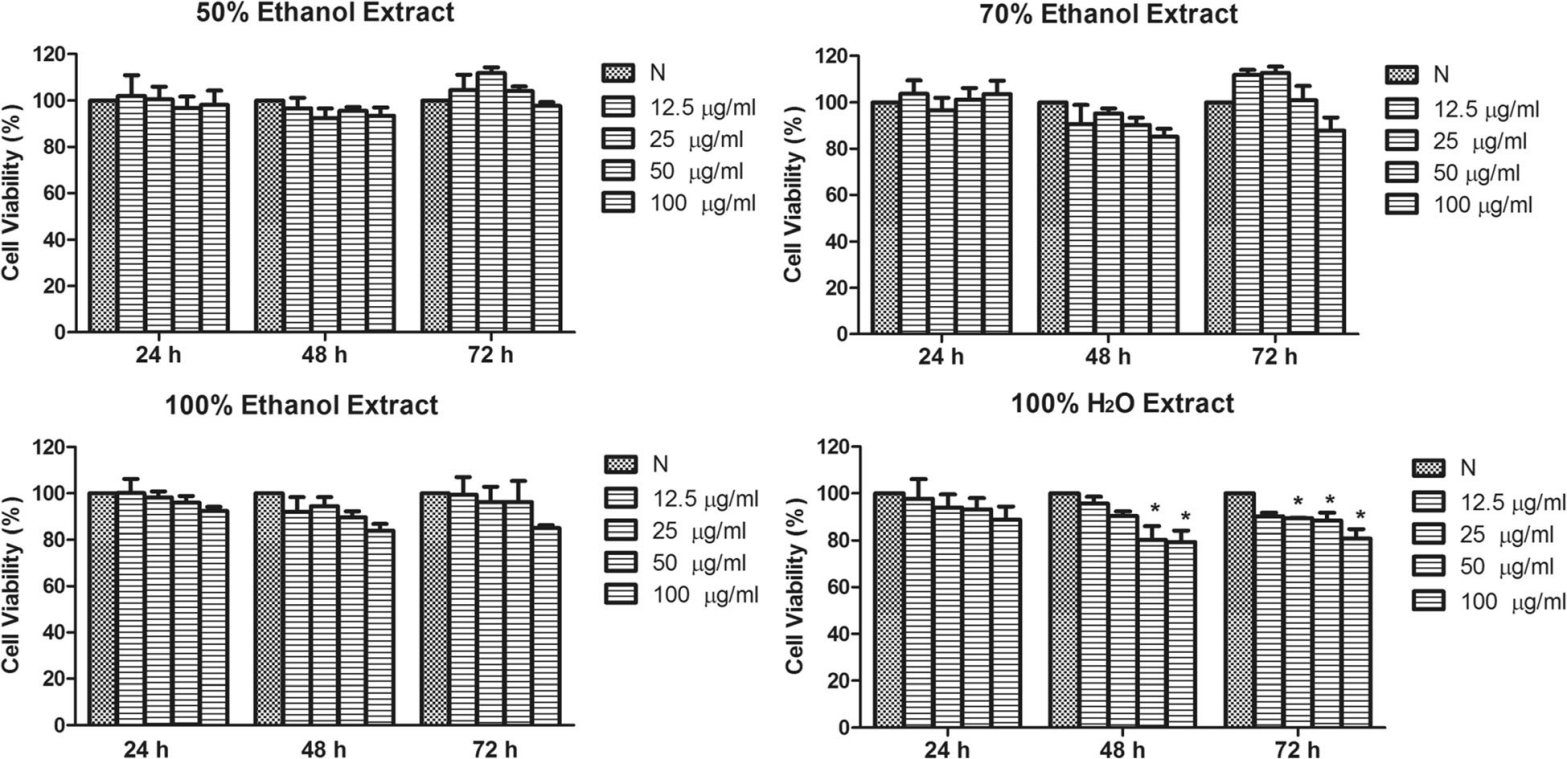
Effects of 50, 70, 100% ethanol extracts and 100% water extract of *C. nutans* leaves on endothelial cell proliferation at 24, 48 and 72 h. The water extract of *C. nutans* leaves was chosen for subsequent experiments. The values presented are the mean ± SEM of three independent experiments and compared against normal control (N). *p < 0.05 were considered significant

Extraction of *C. nutans* using 50, 70 and 100% ethanol did not affect HUVEC viability regardless of its concentrations up to 100 μg/ml. Such effect remains unaltered even with prolonged treatment time from 24 to 72 h (Fig. 1). On the contrary, 50 and 100 μg/ml of *C. nutans* in water extract showed significant reduction of HUVEC viability to 80.20 ± 5.82 and 79.33 ± 4.81%, respectively, after 48 and 72 h as compared to untreated control. At a lower *C. nutans* concentration of 25 μg/ml, such significant difference was only observed after 72 h (Fig. 1). These data suggest that the water soluble compounds within the *C. nutans* water extract could suppress endothelial cell growth. We then tested the *C. nutans* water extract at higher concentrations ranging from 50 to 1000 μg/ml to see if the anti-proliferative effect can be seen within 24 h without causing significant cytotoxicity. However, water extract of *C. nutans* neither suppressed HUVEC growth, nor caused cell death after 24 h (data not shown).

**Fig. 2:**
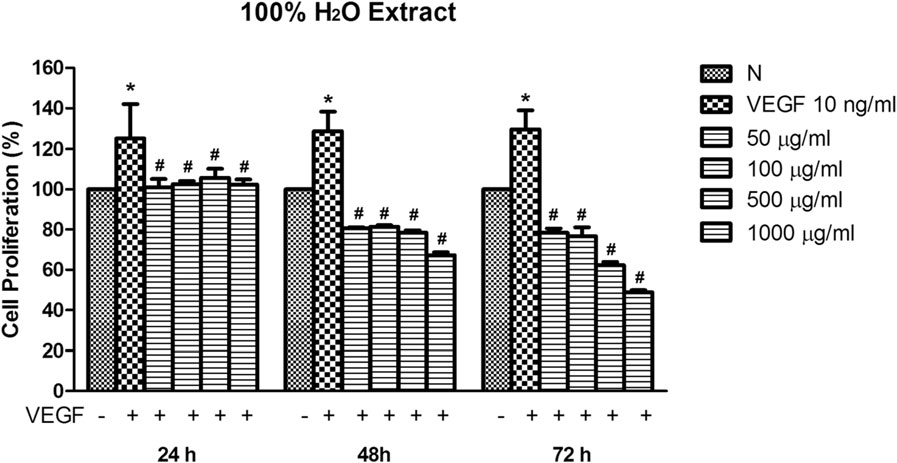
Effect of *C. nutans* water extract on VEGF-induced HUVECs proliferation. HUVECs were co-treated with 50–1000 μg/ml *C. nutans* water extract and 10 ng/ml VEGF for 24, 48 and 72 h. The values presented are the mean ± SEM of three independent experiments and compared against normal control (N). ^*^p < 0.05 were considered significant when compared to normal control (N). # *p* < 0.05 were considered significant when compared to VEGF alone group

VEGF is the known driver of endothelial cell proliferation within tumour microenvironment condition [18]. To better mimic the pathologic conditions, we treated the HUVEC with the water extract of *C. nutans* in the presence of VEGF for 24, 48 and 72 h. As expected, with increasing concentration of *C. nutans* from 50 to 1000 μg/ml, the water extract significantly suppressed VEGF-induced HUVECs proliferation from 80.72 ± 0.42% to 67.25 ± 1.42% (*p* < 0.05) in 48 h, and 78.37 ± 2.09 to 48.88 ± 0.93% in 72 h (*p* < 0.05) as compared to VEGF alone (Fig. 2). These findings suggest the water extract could suppress endothelial growth in dose-dependent manner.

### *C. nutans* water extract suppresses endothelial cell migration

**Fig. 3:**
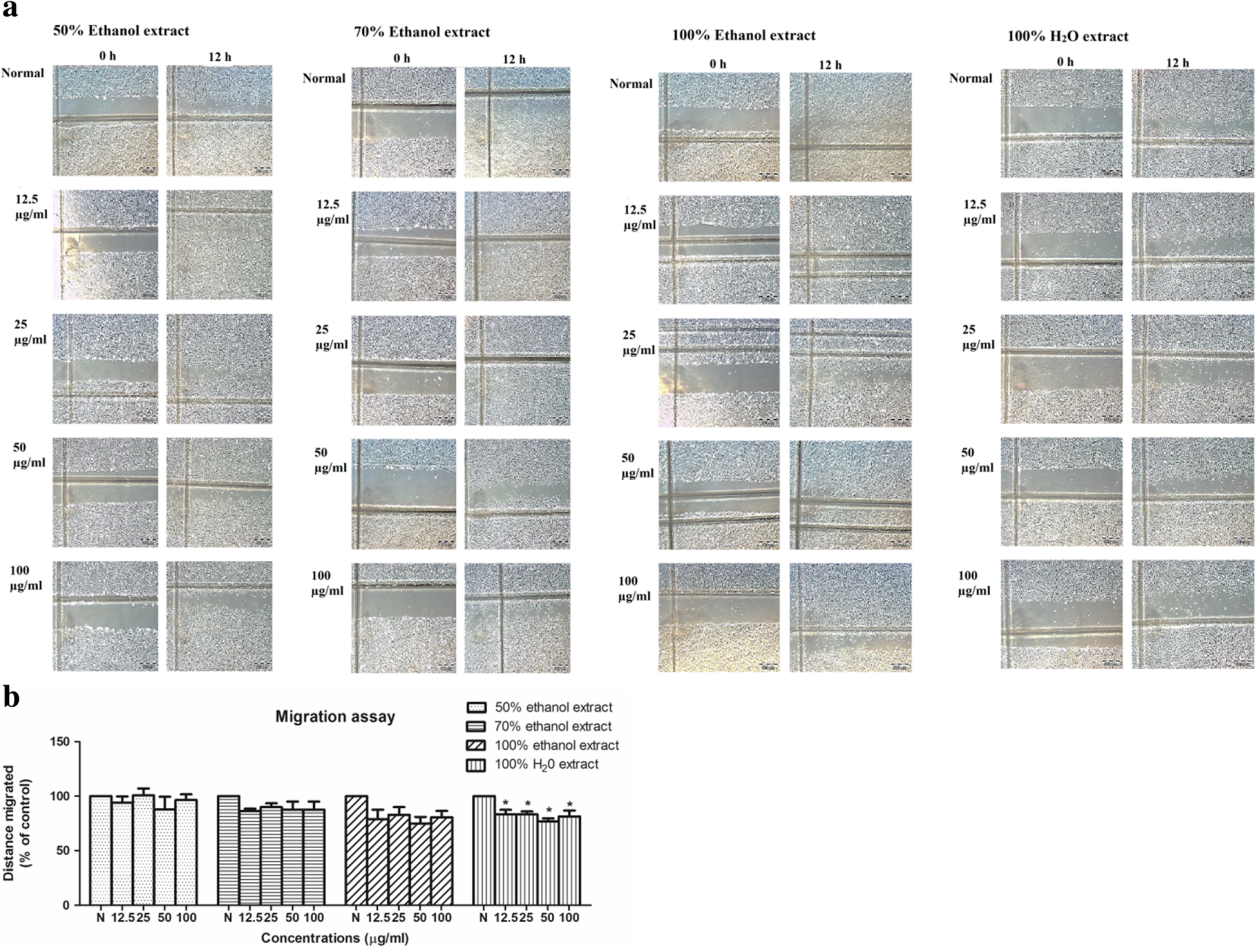
Effects of 50, 70 and 100% ethanol extracts and 100% water extract of *C. nutans* leaves on cell migration. **a** Anti-migratory effects of 50, 70, 100% ethanol extract and 100% water extract of *C. nutans* leaves. Representative width was photographed. **b** Water extract of *C. nutans* leaves is capable to reduce HUVEC migration rate. The values presented are the mean ± SEM of three independent experiments and compared against normal control (N). *p < 0.05 were considered significant

Next, we examined the effects of ethanol and water extract of *C. nutans* on endothelial cell migration based on wound-healing assay. Again, no inhibitory effect was observed in ethanol extract-treated group. However, when HUVECs were treated with the *C. nutans* water extract with increasing concentrations from 12.5 μg/ml to 100 μg/ml, the migration distance was reduced from 83.21 ± 4.24% to 81.50 ± 5.29% (*p* < 0.05) (Fig. 3).

**Fig. 4:**
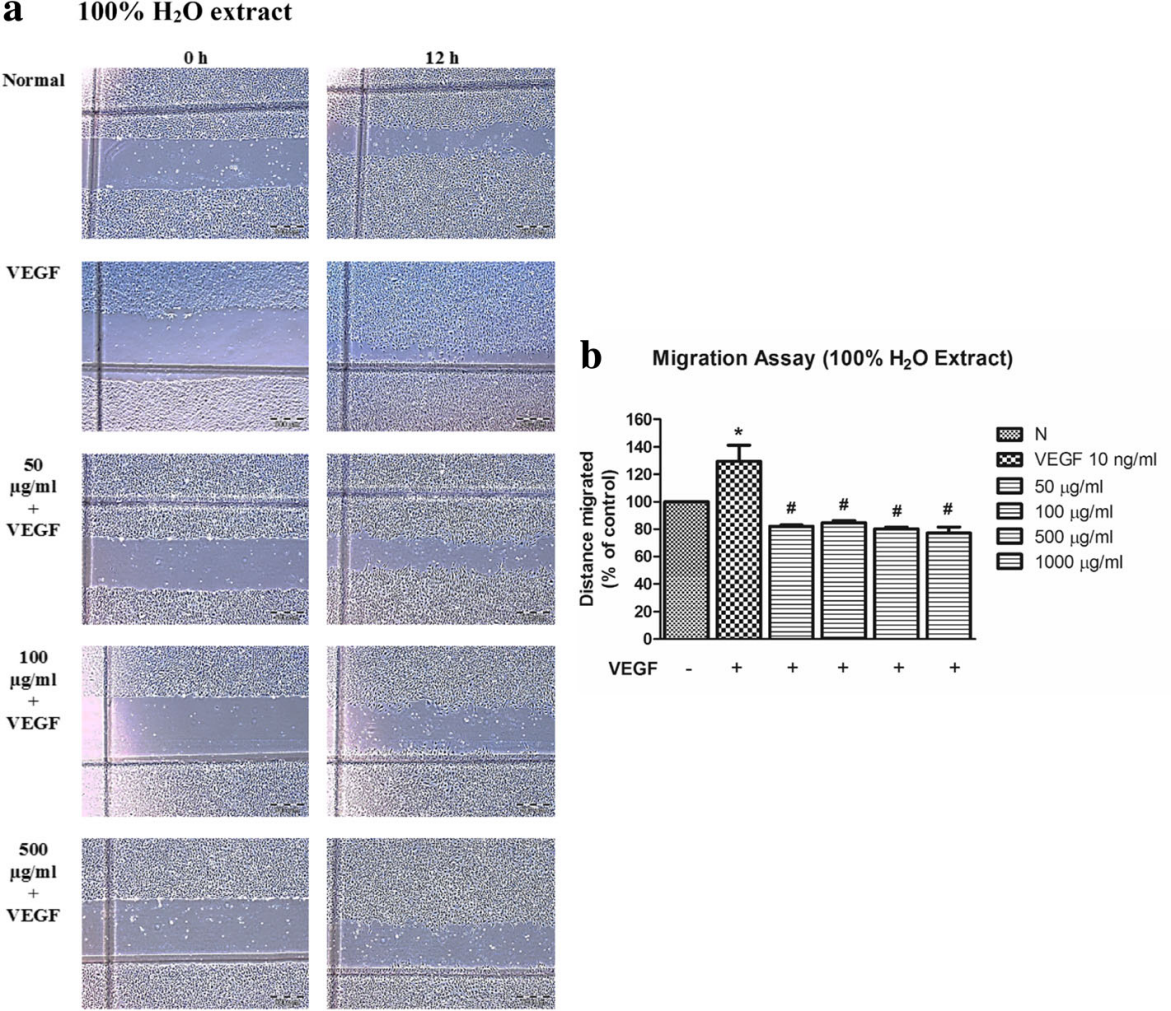
Effect of *C. nutans* water extract on VEGF-induced cell migration. **a** Anti-migratory effect of 100% water extract of *C. nutans* leaves. Representative width were photographed. **b** HUVECs treated with water extract of *C. nutans* leaves showed reduction in migration rate. The values presented are the mean ± SEM of three independent experiments. *p < 0.05 were considered significant when compared to normal control (N); #*p* < 0.05 were considered significant when compared to VEGF alone group

In contrast, HUVECs were also found less migratory when tested with *C. nutans* water extracts at high concentrations from 50 μg/ml to 1000 μg/ml*,* with the measured cell migration distance value significantly reduced from 82.04 ± 0.92% to 77.16 ± 4.33% (*p* < 0.05) (Fig. 4) in the presence of VEGF. These results suggest that water extract of *C. nutans* disrupts VEGF-stimulated HUVEC migration.

### *C. nutans* water extract does not suppress VEGF-induced HUVECs transmigration

**Fig. 5:**
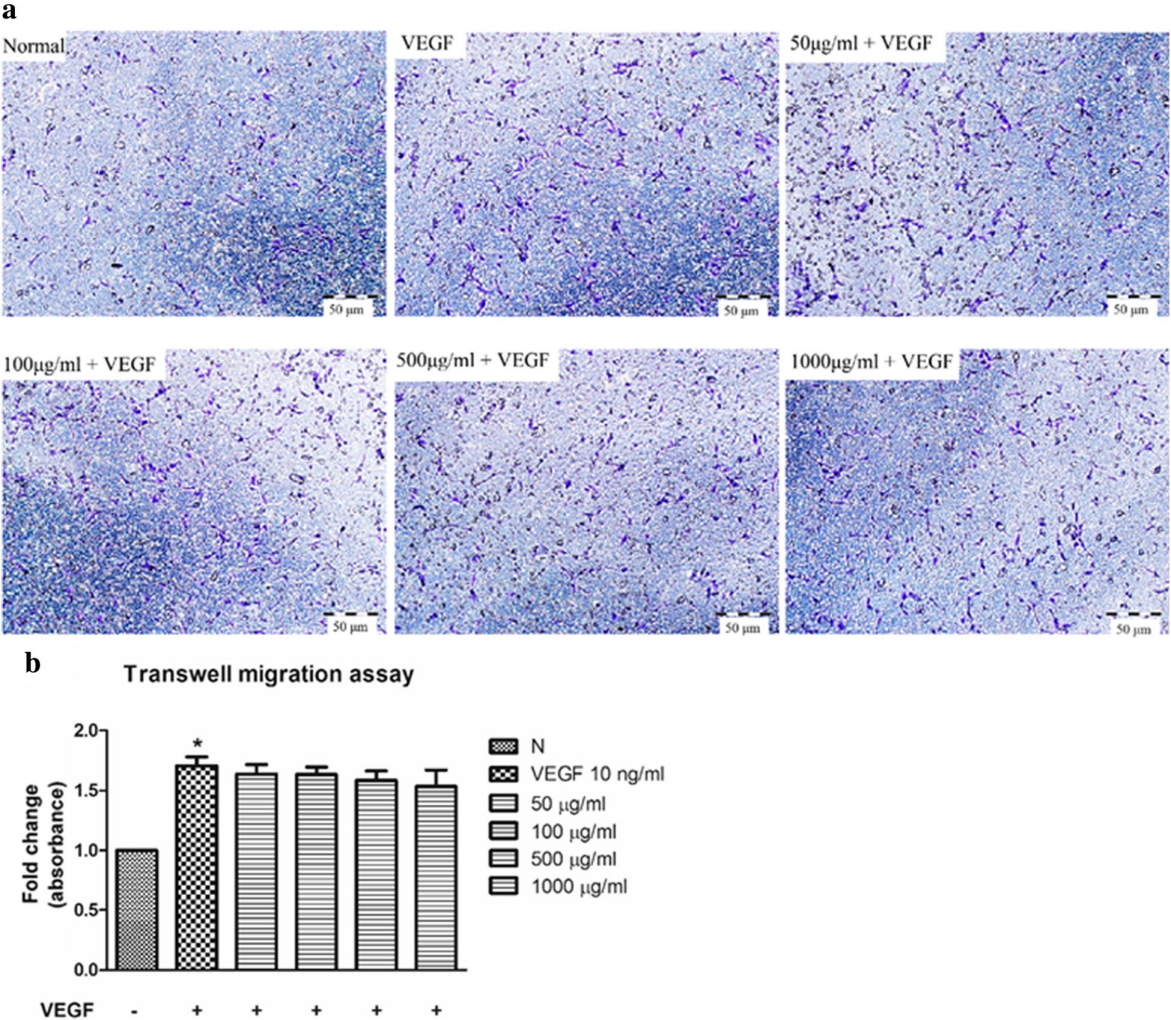
Effect of *C. nutans* water extract on VEGF-induced transwell migration. HUVECs were treated with 50-1000 μg/ml of the water extract for 6 h. **a** The cells were stained with 0.1% crystal violet. Representative results were shown. **b** Quantitative analysis of crystal violet intensities using a microplate reader. The values presented are the mean ± SEM of three independent experiments. *p < 0.05 were considered significant when compared to VEGF alone group

Endothelial cell migration is known to be driven by VEGF gradient [19]. A Boyden chamber assay, which creates VEGF gradient between the upper and lower chambers, was used to investigate effect of *C. nutans* water extract on HUVEC-transmembrane cell migration. As shown in Fig. 5, VEGF induced HUVEC-transmembrane migration by 1.71 ± 0.07 fold as compared to the untreated control. However, this event was not reversed by *C. nutans* water extract, confirming the inability of *C. nutans* water extract in altering HUVEC chemotaxis in response to VEGF.

### *C. nutans* water extract does not suppress VEGF-induced capillary tube formation

**Fig. 6:**
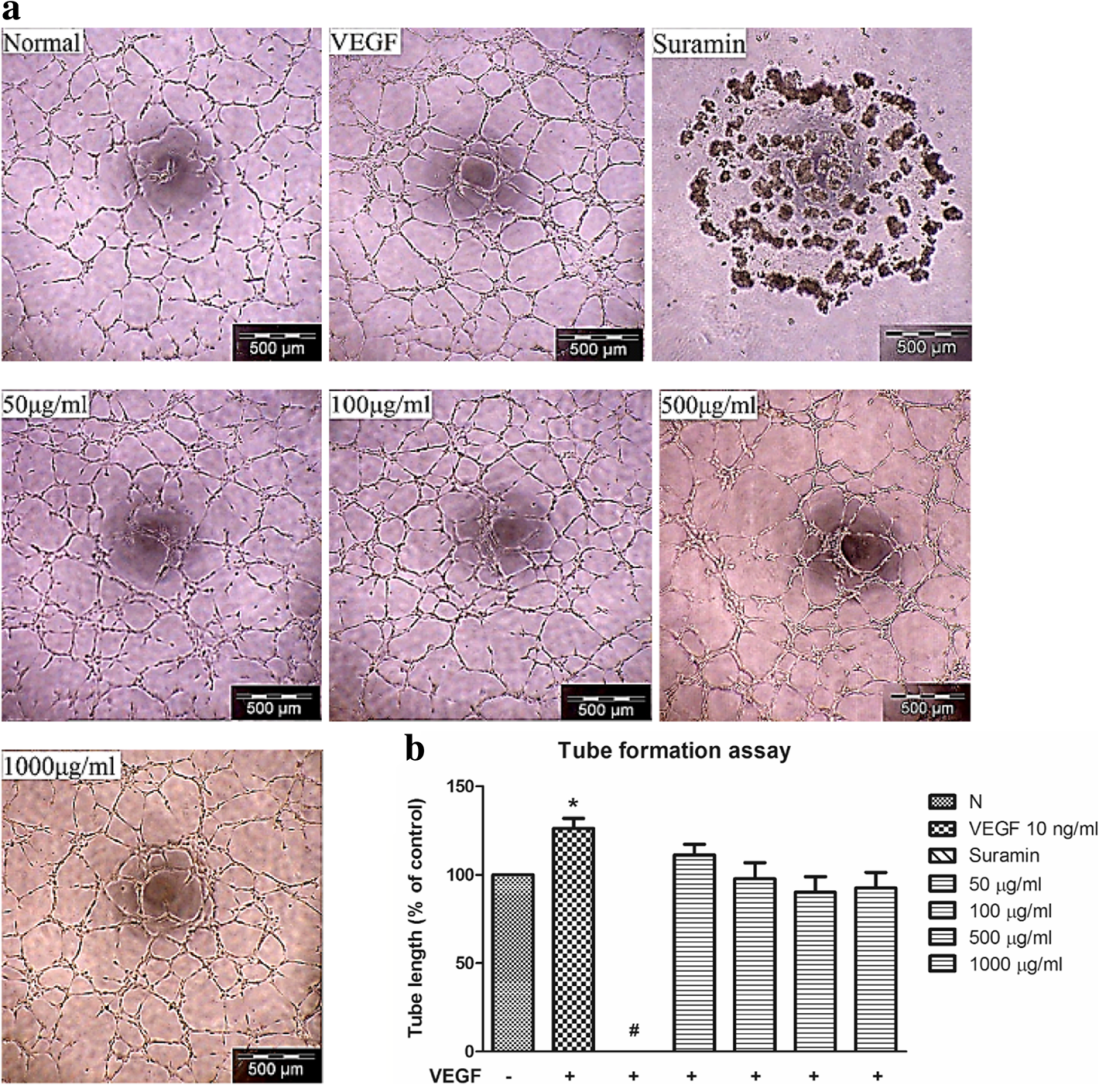
Effect of *C. nutans* water extract on VEGF-induced capillary-like endothelial tube formation. **a** Microscopic examination demonstrated *C. nutans* extract does not suppressed VEGF-induced capillary-tube like formation in comparison with VEGF group. **b** Capillary-tube like vessel density was calculated and compared. The values presented are the mean ± SEM of three independent experiments. *p < 0.05 were considered significant when compared to normal control (N); #*p* < 0.05 were considered significant when compared to VEGF alone group

It is known that a complex endothelial tubular network can be formed when endothelial cells were cultured in three dimensions Matrigel bed in the presence of VEGF [20], and such event can be disrupted by Suramin, a drug which is commonly used in treating sleeping sickness and river blindness [21]. Here we showed that VEGF induced HUVEC capillary tube formation in Matrigel after 3 h, with the measured tube length of 126.30 ± 5.64% as compared to untreated control; whereas Suramin treated-HUVECs lost its tube formation capability completely (Fig. 6). To examine if the water extract of *C. nutans* could suppress VEGF-induced endothelial tube formation, we tested the extract at high concentrations from 50 to 1000 μg/ml. However, no suppressive effect was observed in all *C. nutans* treated group.

### *C. nutans* water extract increases endothelial permeability

**Fig. 7:**
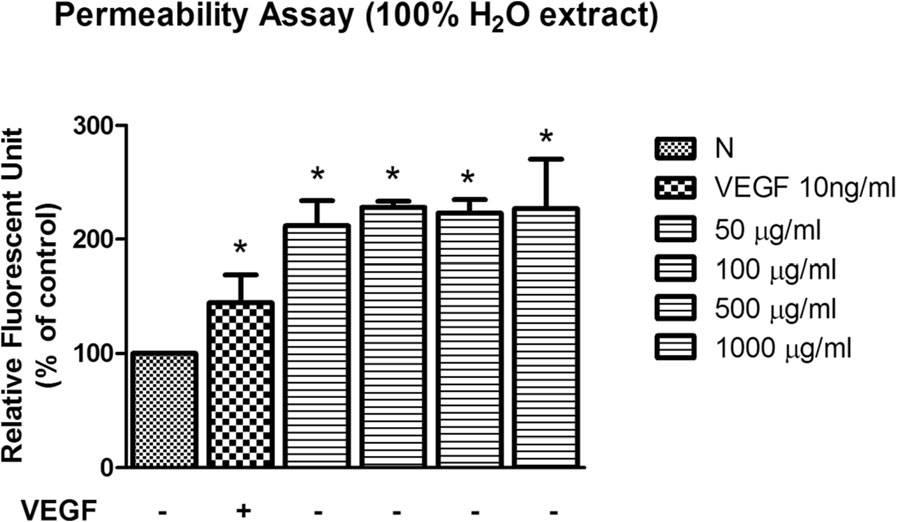
Effect of *C. nutans* leaf extract on endothelial permeability. The values presented are the mean ± SEM of three independent experiments. *p < 0.05 were considered significant when compared to normal control (N)

The endothelial permeability, measured by fluorescent unit relative to the amount of fluorescein isothiocyanate (FITC)-dextran which passes through the cell monolayer, showed that *C. nutans* water extract significantly increased endothelial permeability levels to 211.80 ± 22.03, 228.10 ± 5.23, 223.20 ± 11.80 and 226.80 ± 43.67% when treated at 50, 100, 500 and 1000 μg/ml, respectively (Fig. 7). VEGF was used as a reference whereby VEGF increased HUVEC permeability to 202.27 ± 9.90% of control.

As VEGF secreted by tumor cells plays a crucial role in neoangiogenesis, we also investigate *C. nutans* water extract if it is capable of inhibiting VEGF secretion by human oral squamous cell carcinoma, HSC-4 cells [22]. Nonetheless, all doses of *C. nutans* water extract failed to reduce the VEGF level at 72 h post-treatment (data not shown). These data indicate that *C. nutans* does not attenuate VEGF production by cancer cells.

### *C. nutans* water extract suppresses VEGF-induced aortic ring sprouting and angiogenesis in CAM model

**Fig. 8:**
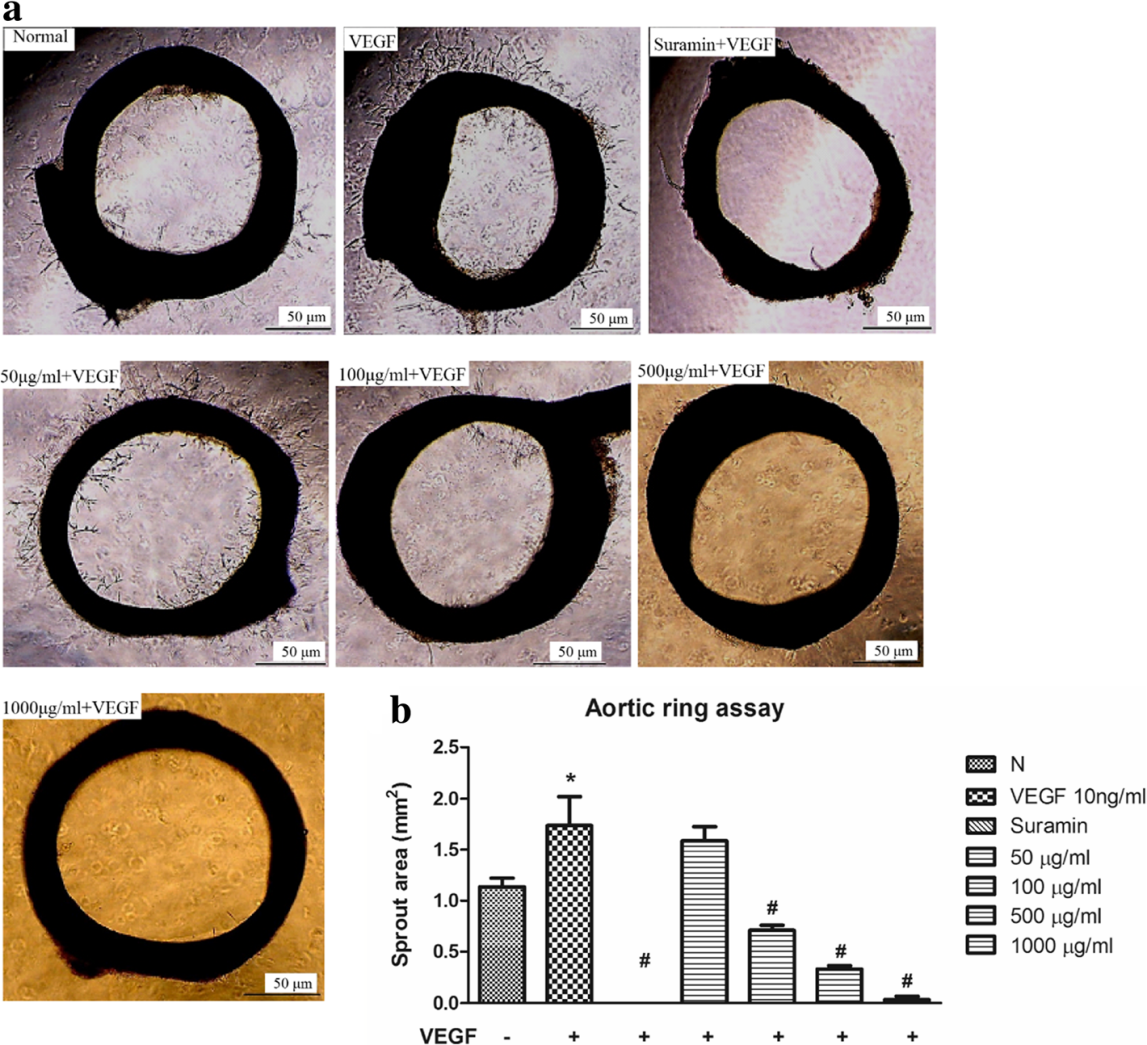
Effect of *C. nutans* leaf extract on VEGF-induced microvessel sprouting of rat aortic ring. Suramin at a dose of 50 μM was used as a positive control. **a** *C. nutans* water extract inhibited vessel sprouting in rat aorta ring induced by VEGF. Representative results were shown. **b** The sprout length was quantified. The values presented are the mean ± SEM of three independent experiments. *p < 0.05 were considered significant when compared to normal control (N); #*p* < 0.05 were considered significant when compared to VEGF alone group. (*n* = 3)

**Fig. 9:**
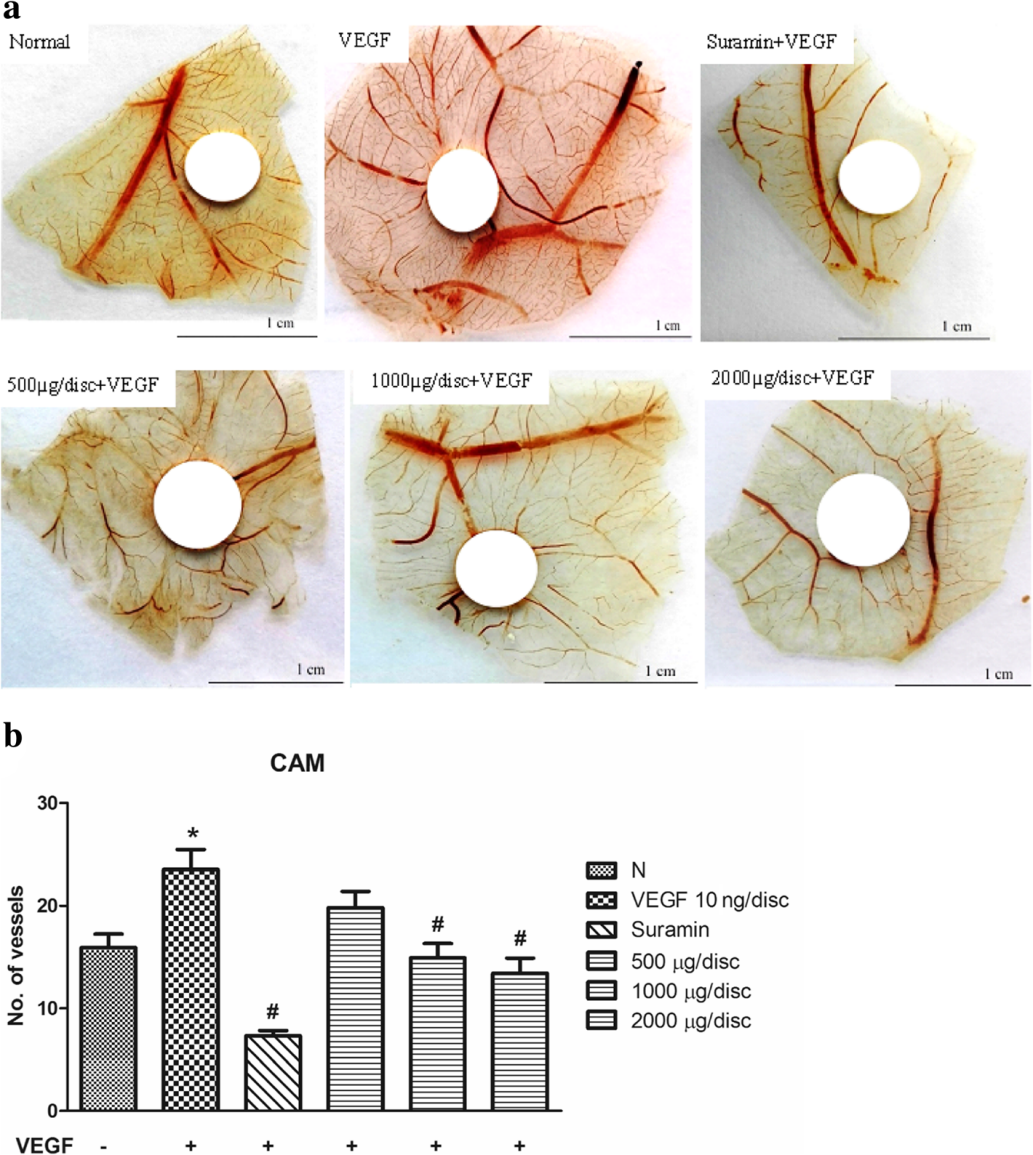
Effect of *C. nutans* water extract on VEGF-induced angiogenesis. Suramin at a dose of 1000 μg/disc was used as a positive control. **a** *C. nutans* extract inhibited vessel sprouting in CAM model induced by VEGF. Representative results were shown. **b** The histogram shows the number of blood vessels contacting the discs. The values presented are the mean ± SEM of three independent experiments. *p < 0.05 were considered significant when compared to normal control (N); #*p* < 0.05 were considered significant when compared to VEGF alone group

To investigate the effect of *C. nutans* water extract on the VEGF-induced vascular sprouting, a rat aortic ring assay was performed. In the control, the non-stimulated aortic rings exhibited a sprout area of 1.14 ± 0.09 mm^2^ (Fig. 8). Similar to the tube formation assay, the aortic ring sprouting effect was improved to 1.74 ± 0.28 mm^2^ in the presence of VEGF, but was completed abolished by 50 μM Suramin. When the rings were treated with 100, 500 and 1000 μg/ml of *C. nutans* water extracts, sprout area was significantly reduced to 0.71 ± 0.05, 0.33 ± 0.03 and 0.03 ± 0.03 mm^2^, respectively. These data indicate that *C. nutans* water extract inhibits vascular sprouting and fusion of neovessels ex vivo. We then tested this effect in an in vivo CAM model, and observed the similar capability in *C. nutans* water extract, of which successfully diminish microvessel counts to 14.90 ± 1.40 and 13.40 ± 1.49 at 1000 and 2000 μg respectively, as compared to VEGF treated (23.50 ± 1.94) and water-treated control group (15.90 ± 1.34) (Fig. 9). These results suggest that *C. nutans* water extract inhibits angiogenesis in vivo.

## Discussion

Tumour angiogenesis is one the main contributing factors that promotes tumour progression. Thus, targeting the blood supply may limit its growth and progression [23]. However, simultaneous targeting of multiple aspects may be required to achieve robust antiangiogenic response. This multi-targeted effect can be achieved by using combinations of drugs, or from natural medicinal herb extracts which possess various active ingredients. *C. nutans* has emerged as a potential regimen for cancer patients as accumulating lay testimonies claimed that *C. nutans* leaves capable to treat various cancer diseases [7]. Whilst more scientific evidence is needed to conclude its therapeutic use, our previous study has also shown that the *C. nutans* leaves exhibiting anti-proliferative and cytotoxicity effect against numbers of cancer cell lines [7]. Here, we demonstrated the potential of *C. nutans* extracts as being the antiangiogenic agent in cancer treatment.

Under physiological conditions, endothelial cell migration is coordinated by cytoskeleton reorganization where protrusions formed by lamellipodia direct the migrating cells, and this is followed by contraction of stress fibres which allows the cell body to move forward [24]. This dynamic and tightly regulated process, however, is disrupted during tumour angiogenesis, resulting in excessive cell migration and proliferation, the pivotal processes that eventually leads to structure tube and microvessel morphogenesis. In this study, we showed that these processes can be inhibited by *C. nutans*, particularly the extracts from the water soluble fraction. We also tested the effect of the extracts and confirmed their efficacy even in the presence of human VEGF_165_, the most abundant and active isoform of VEGF-A monomer [24], which mediates angiogenesis within tumour microenvironment [25]. Taken together, our data support the notion that the water extract of *C. nutans* is capable of inhibiting VEGF-stimulated angiogenesis by targeting endothelial cell proliferation, structure tubes and microvessel formation, but not endothelial migration. Endothelial cell migration also depends on the existence of a VEGF gradient, which serves as an attractant and regulates the motion of the endothelial tip cell [19]. The Boyden chamber assay demonstrated that *C. nutans* water extract does not prevent HUVEC migration towards a chemotatic gradient, indicating that *C. nutans* does not inhibit VEGF-induced chemotaxis (Fig. 5). “Wound healing” and Boyden chamber both are type of assay to determine the migration rate of the endothelial cells, interestingly, “wound healing” assay was inhibited by the extract but Boyden chamber model failed to be inhibited by the extract. Previously reported that “wound repair” model is a multi-step process involving spreading, proliferation and migration events [26, 27], and our data showed that extract significantly suppressed proliferation. This again confirmed that extract inhibits “wound repair” model may via suppression of proliferation process. However, Boyden chamber model is mainly involved in migration process, thus, this indicate extracts not be able to suppress endothelial migration.

Vascular hyperpermeability is a hallmark of acute inflammation, a pathologic event that responsible for oedema and coronary heart diseases [28]. In cancers, chronic vessel hyperpermeability may facilitate leukocyte infiltration into the tumour, promote metastatic spread of cancer cells [29], and impaired efficient drug delivery due to increased interstitial pressure [30]. Drugs which capable to normalize tumour vasculature and attenuates the exaggerated permeability has emerged to be a new concept in anti-angiogenic therapy [31]. Normalization of abnormal vasculature able to increase the efficacy of conventional therapies and decrease the rate of metastasis even though it makes the vasculature to be more efficient for oxygen delivery towards the tumour [31]. Noteworthy, our findings suggest that *C. nutans* water extract could cause an increase in HUVEC permeability without addition of VEGF. The underlying mechanism is unknown; however, this data discouraged further study and development of *C. nutans* water extract as a potential therapeutic agent.

Apart from the cellular systems, we also examined the beneficial effects of *C. nutans* water extract using both ex vivo and in vivo models. In comparison to isolated cell culture, these models are more physiologically relevant because they allow complex cellular interactions to occur. The aortic ring assay is a type of organ culture that permits the interactions of endothelial cells with their surrounding heterotypic cells. In this assay, the endothelial cells are in a quiescent and non-proliferative state at the time of explantation. In response to pro-angiogenic agents, the cells proliferate, migrate and differentiate into tubular networks, resembling the in vivo condition [32]. In the present study, we found that *C. nutans* water extract significantly inhibits the microvessel outgrowth triggered by VEGF (Fig. 8), suggesting that the water extract of *C. nutans* leaves also possesses potential anti-angiogenic properties in isolated aortic tissues. The CAM model was used due to easy accessibility and highly vascularized nature of the system [33]. We demonstrated that *C. nutans* water extract prevents new blood vessel formation in the CAM model (Fig. 9). This finding is in line with the inhibitory effects of *C. nutans* on cell proliferation and migration observed in vitro, implying that the anti-angiogenic effects of *C. nutans* in HUVECs can be translated to in vivo models as well. GC-MS, chemical, ^1^H NMR and ^13^C NMR, HPLC with tandem mass spectrometry (LC/MS/MS) showed that vitexin, isovitexin and betulin [34] were among the phytochemicals that present in *C. nutans* extract which had previously demonstrated anti-angiogenesis activities [35–37]. Thus, the observed angiogenic activities in this study were likely the effect of, but not limited to, these active compounds, albeit further in-depth investigation is needed.

Number of angiogenic growth factors are known to be involved in the process of angiogenesis, this including VEGF, fibroblast growth factors, and the platelet-derived endothelial cell growth factor [38]. Binding of these angiogenic growth factors to its corresponding receptors on the surface of normal endothelial cell promote the activation of endothelial cells [39]. As a result, new blood vessels sprout from the pre-existing blood vessels. Anti-angiogenic therapies targeting various steps in this process. For instance, bevacizumab specifically binds to VEGF and thus inactivate VEGF [40]. On the other hand, Sunitinib malate has been shown to be a potent inhibitor of VEGF receptors [41]. In the current data, *C. nutans* water extract does not inhibit VEGF secretion by cancer cells**.** This may indicate that *C. nutans* water extract is specific to inhibit the action of VEGF on endothelial cells but not the production of VEGF from the cancer cells. Further investigation is needed in order to confirm whether *C. nutans* water extract is acting through the VEGF receptor.

## Conclusion

In conclusion, we showed that *C. nutans* is capable of impeding VEGF-induced blood vessel formation by targeting on endothelial cell proliferation, aortic vessel sprouting and the growth of micro-vessels in CAM. For the first time, we demonstrate the anti-angiogenic potential of *C. nutans* in water extract and this activity may be associated with the active compounds such as vitexin, isovitexin and betulin. However, more in-depth investigations to identify the molecular target for therapeutic intervention and to evaluate the efficacy and safety of *C. nutans* water extract in diseased animal models are required to warrant translation to therapeutics for clinical use.

## Abbreviations

CAM: Chorioallantoic membrane
FITC: Fluorescein isothiocyanate
HUVECs: Human umbilical vein endothelial cells
VEGF: Vascular endothelial growth factor
